# Prevalence and factors associated with erectile dysfunction among adult men in Moshi municipal, Tanzania: community-based study

**DOI:** 10.1186/s12610-020-00118-0

**Published:** 2020-12-02

**Authors:** Kenan B. Nyalile, Emmanuel H. P. Mushi, Epiphania Moshi, Beatrice J. Leyaro, Sia E. Msuya, Orgeness Mbwambo

**Affiliations:** 1 Institute of Public Health, Department of Community Health, Kilimanjaro Christian Medical University College (KCMUCo), Po Box 2240, Moshi, Tanzania; 2 Institute of Public Health, Department of Epidemiology & Biostatistics, Kilimanjaro Christian Medical University College (KCMUCo), Moshi, Tanzania; 3 Department of Community Health, Kilimanjaro Christian Medical Centre (KCMC), Moshi, Tanzania; 4 Department of Urology, KCMUCo & Kilimanjaro Christian Medical Centre (KCMC), Moshi, Tanzania

**Keywords:** Dysfonction érectile, Diabète sucré, Hypertension, Activités physiques, Prévalence, Facteurs prédictifs, Tanzanie, Erectile dysfunction, Diabetes mellitus, Hypertension, Physical activities, Prevalence, Predictors, Tanzania

## Abstract

**Background:**

Erectile dysfunction (ED) has a negative impact on ones’ relationships with poor quality of life as inevitable result. The effects of ED maybe worse in developing countries setting like Tanzania because men’s sexual health has been forgotten. Men’s sexual and reproductive health needs are not in the national reproductive health strategic. This study aimed to determine the prevalence and factors associated with erectile dysfunction among adult men in Moshi municipality, northern Tanzania.

**Results:**

The mean age of the 381 men was 39.6 (SD ±16.8) years. The overall prevalence of ED on this study was 29.7%. The severity of ED among study participants was; 13.4% (51), 9.7% (37), 3.7% (14), 2.9% (11) had mild, mild to moderate, moderate and severe erectile dysfunction respectively. Age 40–54 years (Adjusted OR 5.0, 95% CI 2.5–9.9), > 55 years (aOR 11.7, 95% CI 5.8–23.7) and hypertension (aOR 2.6, 95% CI 1.1–6.4) were independent predictors of ED respectively.

**Conclusion:**

The prevalence of ED is high among men in Moshi municipal as 1 out of 3 men have ED. Age and hypertension were independent predictors of ED. These results point to the need of community awareness and education programs to raise awareness among men about existence of ED problem, its consequence and where they can get advice and care in this setting. Further, health providers taking care of hypertensive and men with DM should be equipped with knowledge and skills on early detection for ED and how to counsel and where to refer patients for help.

## Résumé

**Contexte:**

La dysfonction érectile (DE) a un impact négatif sur les relations, avec une qualité de vie moindre comme résultat inévitable. Les effets de la DE peuvent être pire dans les pays en développement comme la Tanzanie parce que la santé sexuelle des hommes a été oubliée. Les besoins des hommes en matière de santé sexuelle et reproductive ne sont pas inclus dans le cadre de la stratégie nationale en matière de santé reproductive. Cette étude avait pour objectif de déterminer la prévalence et les facteurs associés à la dysfonction érectile chez les hommes adultes dans la municipalité de Moshi, dans le nord de la Tanzanie.

**Résultats:**

L’âge moyen des 381 hommes était de 39,6 ans (SD 16,8). La prévalence globale de la DE dans cette étude était de 29,7%. La sévérité de la DE chez les participants à l’étude a été comme suit: 13.4% (51) avaient une dysfonction érectile légère, 9,7% (37) une DE légère à modérée, 3,7% (14) une DE modérée, et 2,9% (11) sévère. Les facteurs de prédiction indépendants de la DE étaient respectivement un âge entre 40–54 ans (OR ajusté = 5,0; IC 95% 2,5-9,9), un âge > 55 ans (OR ajusté = 11,7; IC 95% 5,8-23,7), et la présence d’une hypertension (OR ajusté = 2,6; IC 95% 1,1-6,4).

**Conclusions:**

Il existe une prévalence élevée de la DE chez les hommes dans la municipalité de Moshi, avec 1 homme sur 3 qui présente une DE. L’âge et l’hypertension étaient des facteurs de prédiction indépendants de la DE. Ces résultats indiquent un besoin de programmes de sensibilisation et d’éducation communautaires pour sensibiliser les hommes à l’existence d’un problème de DE, de ses conséquences et des lieus où ils peuvent obtenir des conseils et des soins dans ce cadre. En outre, les prestataires de soins de santé qui s’occupent d’hommes hypertendus ou d’hommes atteints de diabète sucré devraient avoir des connaissances et des compétences sur la détection précoce de la DE, sur la façon de conseiller ces hommes et de les informer des lieux où ils peuvent trouver de l’aide.

## Background

Erectile dysfunction (ED) is defined as the persistence inability to achieve and maintain an erection adequate to permit satisfactory sexual act [1]. Though considered by many to be part of normal aging process, ED can be an outcome of several causes. ED has been found to be more prevalent among aged population [2], smokers [3] and in population with comorbidities like diabetes mellitus [4], peripheral vascular disorders, coronary artery disease [5] and among individuals with hypertension [6]. Psychogenic illness like anxiety [7], and depression [8] have also shown an impact on ED.

In Africa, few epidemiological studies have been conducted concerning the burden of erectile dysfunction. Studies of ED in Africa have been done both at community and hospital settings. The prevalence of ED in a population-based study in Egypt was 10% for moderate and 13% for severe ED, while in Ghana a study among men aged 19 years or more in a population reported prevalence of 65.9% [3, 9]. In Nigeria the prevalence of ED ranged from 43.8–58.9% in a community-based setting [2, 10], without much difference (41.5–65.8%) with ED prevalence among men attending hospitals for diabetic, hypertension or outpatient care [6, 11]. In Tanzania a study in Dar es Salaam, showed ED prevalence of 24 and 55% among men in a community-setting and those attending at diabetic care respectively [4, 12]. Nearly all the studies used men aged 18 years and above and cross-section design.

ED has a negative impact on men’s health. Poor quality of life has been described in men with ED. Negative consequence on men’s mental health like depression, anxiety and low self-esteem have also been reported [8, 13]. The effects of ED maybe worse in developing countries setting like Tanzania because men’s sexual health has been forgotten. Men’s sexual and reproductive health needs are not in the national reproductive health strategic plans [14]. Studies have shown an association between ED with advanced age, smoking, and presence of hypertension and diabetes [3, 10, 12]. Given differences on ED prevalence within the same country, maybe due to differences in background occurrence of co-morbidities like diabetes and hypertension among men, there is a need to have information from different settings and population to understand the burden of the problem. This community-based study was conducted to determine the prevalence and factors associated with ED in an urban setting in northern Tanzania.

## Methods

### Study design and site

The study was a community- based cross sectional study. Recruitment and data collection were conducted in July 2019, at Moshi Municipal, situated in Kilimanjaro region in the northern Tanzania.

Kilimanjaro is one of the 31 regions found in northern zone of Tanzania. It is the home of 1.64 million people according to 2012 census. Moshi municipal is one of the seven district of Kilimanjaro region. It has 21 wards and covers an area of 59 km^2^. The municipal has a population of 184,292 where males are 89,174 and females are 95,118 according to 2012 census.

### Study population, sampling and data collection procedures

#### Population

The study population was men aged at least 18 years in selected wards of Moshi Municipal and consented to participate in the study. The study excluded men who are not permanent residents in the study area.

#### Sample size

Sample size for this study was estimated by using the formula for precision

$$ \mathrm{N}={\mathrm{Z}}^2\mathrm{p}\ \left(1-\mathrm{p}\right)/{\upvarepsilon}^2 $$

Where ***N*** is estimated minimum sample size; ***Z*** is confidence level at 95% (standard value is 1.96); ***P*** is proportion of ED in population based on study by Pallangyo et al., where the prevalence of ED was 24% [12] and ***ε*** is precision at 95% CI = 0.05.

The minimum sample that was required for this study was 346 men. Addition of 10% for non-response gave a minimum sample of 381 men aged at least 18 years.

#### Sampling

Multistage sampling technique was used to obtain men who participated in the study. The multistage sampling techniques involved the following stages. Stage 1: 4 out of 21 wards that make up the municipal were randomly selected. Stage 2: Two streets from each of the selected ward were randomly selected: At Majengo ward, Shauri moyo and Sokoni streets, were randomly selected. At Mji Mpya ward, Sokoni and Langoni streets were randomly selected. At Kiboriloni ward, mnazi mmoja and Kilimanjaro District Council (KDC) streets were randomly selected. At Rau ward, Saba saba and Karikacha streets were randomly selected. From these streets, men aged 18 years and above who consented to participate in this study were recruited.

#### Study procedures

Ethical clearance was obtained from the Kilimanjaro Christian Medical University College (KCMUCo) Ethical committee before starting the research. The ethical clearance was delivered to District Medical Officer (DMO) of Moshi Municipal, where permission to conduct the study was sought from the DMO office. DMO gave us introductory letter that was presented to Ward and streets leaders. The ward and street leaders were all informed about their key role of introducing researchers to the community and help to inform adult males about the study prior to the beginning of the study.

After reaching to the center which was established, at respective wards those who were eligible and consented to participate were interviewed. The interviews were conducted by three medical students who were in their 4th year of training and have rotated in the urology department for four weeks. They underwent training by the consultant urologist (OM) for 3 days before commencement of data collection. This was followed by general clinical examination and measurement of the blood pressure (BP), anthropometric measurements and taking blood for Random blood glucose level (RBG) measurement. The interviews and clinical examination were conducted in private room which was either a ward executive officer office or room at a local clinic. After the test participants were informed the results of BP, overweight/obesity and RBG on the same day.

Those found with confirmed either diabetes or hypertension were informed about their results and then referred to the nearby health centre. Those with erectile dysfunction were referred at KCMC referral hospital urology clinic where there is expertise to manage the condition. The physician responsible was then informed about their health status through a referral letter. They were also given advice on diet, physical activity, on salt and alcohol intake.

### Data collection tool and methods

#### Data collection tools

Data was collected using a questionnaire which had both open and closed ended questions and was in Swahili language. The questionnaire had three sections, the first part collected information on socio-demographic characteristics, and the second part assessed the behavioural characteristics of the participant. The third part was clinical examination. The validated Swahili-translated 5-item version of the international index of erectile function (IIEF-5) scale was used to assess erectile dysfunction.

#### Data collection methods

Face to face interview was used to collect information from the consented participant. Blood pressure was measured twice before and after the interview. Weight (kg), height (cm), blood sample (finger prick) was collected to check for random blood glucose (RBG). Data collection process took place privately in a selected room (i.e. ward executive office).

### Anthropometric measurement

Weight in kg was measured using weighing machine (SECA brand) participants were requested to remove shoes, extra clothing like jackets and anything in pockets before measurements. Height was measured with a height stadiometer where by individual stood upright with no shoes and the ruler hook was placed such that it just touches the head then the height in cm was recorded. Body Mass Index (BMI) was calculated by a ratio of weight (in kilograms) to height (in meters) squared. BMI cut-off values of World Health Organization (WHO) were used.

### Blood pressure measurements

Blood pressure was measured by manual BP machines. BP was recorded from the left arm using an appropriate size cuff that covers two – third of the upper arm after the participant rested for at least five minutes and no smoking or caffeine 30 min before measurements, with the arm supported at the level of the heart, palm facing upward. Two measurement were taken at the start of interview and at the end in a sitting position while the participant rested quietly, then the average of the two measurement was recorded.

### Blood glucose measurements

Random Blood glucose was measured using a blood glucose monitor (GlucoPlus brand). Blood sample was collected through finger pricking and dropped on glucometer strip to give the reading.

### Data analysis

The data was entered and analysed using SPSS 20. Before analysis, data was cleaned by running frequency of each variable. Categorical variables were summarized into frequency and percent while continuous variables were summarized using measures of central tendency and their respective measure of dispersion. Odds ratio with their 95% confidence interval were used to measure the strength of association between age, alcohol drinking, tobacco use, physical activities, overweight/obesity, hypertension, diabetes mellitus and erectile dysfunction. Multivariable logistic regression analysis was conducted to control for confounders and get independent predictors for erectile dysfunction. *P*-value of < 0.05 was considered statistically significant.

### Categorization of variables

ED was assessed using IIEF with 5 questions each with scale of 5. Maximum possible score is 25 and minimum score is 5. The IIEF-5 scale categorizes ED into five categories depending on the score i.e. 22–25: no ED, 17–21: mild ED, 12–16: mild to moderate ED, 8–11: moderate, 5–7: severe ED.

A systolic blood pressure (SBP) < 120 mmHg and a diastolic blood pressure (DBP) < 80 mmHg was defined as normotensive. Pre- hypertension was defined as SBP of 120–139 mmHg or DBP of 80–89 mmHg, while SBP ≥140 mmHg or DBP ≥90 mmHg indicated hypertension.

Diabetes was defined as RBG ≥11.1 mmol/L. Prediabetes was defined as RBG of 7.8–11.0 mmol/L.

WHO BMI cut-off values were used. Underweight was defined as BMI of < 18.5 Kg/m^2^, normal was defined as BMI of 18.5–24.9 Kg/m^2^, overweight was defined as BMI of 25–29.9 Kg/m^2^ and obese was defined as BMI of ≥30 Kg/m^2^.

Physical inactivity was defined as having less than 150 min of moderate to vigorous -intensity physical activity throughout the week.

## Results

### Socio-demographic, life style and clinical characteristics of participants

A total of 390 men aged 18 years and above were approached and 381 (97.7%) agreed to participate in the study. The age of the participants ranged from 18 to 87 years with mean age of 39.6 (±16.8) years. Of the 381 participants; 61.2% were married/cohabiting, 52.0% had secondary education or higher, 49% reported to be taking alcohol, 22.0% were smoking cigarette and 36% had < 150 min of moderate to vigorous intensity physical activities per week, Table 1.

**Table 1:** Socio-demographic, life style and clinical characteristics of study participants (*N* = 381)

Characteristic	Frequency	%
**Age group (years)**		
18–39	227	60
40–54	67	17
≥ 55	87	23
**Education level**		
No formal education	8	2
primary education	175	46
Secondary education/high	198	52
**Marital status**		
Single	127	33
Married/cohabiting	233	61
Divorced/widower	21	6
**Alcohol intake**		
Yes	185	49
No	196	51
**Tobacco use**		
Yes	84	22
No	297	78
**Physical activities**		
None	71	19
< 150 min/week	65	17
≥ 150 min/week	245	64
**Body mass index (kg/m**^**2**^**)**		
Normal^a^	215	56
Underweight^b^	28	7
Overweight^c^	101	27
Obesity^d^	37	10
**Blood pressure (mmHg)**		
Normal^x^	246	65
Pre-hypertensive^y^	88	23
Hypertension^z^	47	12
**Blood sugar level (mmol/L)**		
Normal^k^	354	93
Pre diabetes^l^	19	5
Diabetes^m^	8	2

^a^ 18.5–24.9 kg/m^2^, ^b^ < 18.5 kg/m^2^,^c^ 25–29.9 kg/m^2^,^d^ ≥ 30 kg/m^2^

^x^ < 120/80 mmHg ^y^ 120–139/89-89 mmHg ^z^ ≥ 140/≥90 mmHg

^k^ < 7.8 mmol/L ^l^ 7.8-11 mmol/L ^m^ ≥ 11.1 mmol/L

The mean BMI was 24.4 (±10.3) kg/m^2^ and 36.2% were overweight and obese. The mean SBP and DBP were 122 (±29) mmHg and 77 (±10) mmHg respectively and 12.3% of the participants had high blood pressure. The mean random blood glucose level was 5.9 (±1.5) mmol/L and 2.1% of the 381 men had diabetes mellitus (Table 1).

### Prevalence and factors associated with erectile dysfunction(ED)

The overall prevalence of erectile dysfunction among adult males in Moshi municipal was 29.7% (*n* = 113). Of the 113 men with ED, 45.3% (51) had mild form, 32.7% (37) had mild to moderate form, 12.3% (14) had moderate form and 9.7% (11) had severe form.

In bivariate analysis, age, alcohol use, overweight and obesity, high blood pressure and pre-diabetes/diabetes were associated with erectile dysfunction (Table 2). Participants aged 40–54 and ≥ 55 had 6.6 and 16.0 higher odds of having ED compared to participants aged 18–39. Men who were overweight had 90% higher odds of ED and obese had 2-fold odds of ED compared to men with normal BMI. Pre-hypertensive and hypertensive men had 80% higher odds of ED than to normotensive men and men with pre diabetes/ diabetes had 5-fold higher odds of ED than men with normal values.

**Table 2:** bivariate and multivariable logistic regression model for factors associated with erectile dysfunction (*N* = 381)

Variable	ED = 113	cOR (95% CI)	aOR (95% CI)
	n (%)		
**Age (years)**			
18–39	25 (11.0)	1	
40–54	30 (44.8)	6.6 (3.5–12.4)	5.0 (2.5–9.9)*
≥ 55	58 (66.7)	16 (8.8–29.7)	11.7 (5.8–23.7)*
**Alcohol intake**			
No	55 (28.1)	1	
Yes	58 (31.8)	1.2 (0.8–1.8)	
**Tobacco use**			
No	80 (27.1)	1	
Yes	33 (38.4)	1.7 (1–2.8)	1.5 (0.8–2.9)
**Physical activity**			
≥ 150 min/week	69 (28.2)	1	
None/< 150 min/week	44 (32.4)	1.2 (0.8–1.9)	
**Body mass index (kg/m**^**2**^**)**			
Normal^a^	53 (24.7)	1	
Overweight^c^	39 (38.6)	1.9 (1.2–3.2)	1.5 (0.8–2.9)
Obesity^d^	15 (40.5)	2.1 (1–4.3)	1.4 (0.6–3.6)
**Blood pressure (mmHg)**			
Normal^x^	52 (21.1)	1	
Pre-hypertensive^y^	29 (33.0)	1.8 (1.1–3.2)	1.2 (0.6–2.2)
Hypertensive^z^	32 (68.1)	8.0 (4–15.8)	2.6 (1.1–6.4)*
**Blood sugar level (mmol/L)**			
Normal^k^	95 (26.8)	1	
Prediabetes^l^/Diabetes^m^	18 (66.7)	5.5 (2.4–12.6)	2.5 (0.9–6.6)

Adjusted for Age, Tobacco use, Body mass index, Blood pressure and Blood sugar level

*CI* Confidence interval, *cOR* Crude odds ratio, *aOR* Adjusted odds ratio, *ED* Erectile dysfunction *statistically significant

^a^ 18.5–24.9 kg/m^2^, ^c^ 25–29.9 kg/m^2^, ^d^ ≥ 30 kg/m^2^

^x^ < 120/80 mmHg, ^y^ 120–139/89-89 mmHg, ^z^ ≥ 140/≥90 mmHg

^k^ < 7.8 mmol/L, ^l^ 7.8-11 mmol/L, ^m^ ≥ 11.1 mmol/L

In multivariable logistic regression analysis, age and high blood pressure remained associated with ED. Men aged 40–54 years and those 55+ had 5.0 and 11.7 significantly higher odds of ED than men aged 18–39 years. Likewise, men who were hypertensive had 2.6 higher odds of having ED than those who had normal blood pressure, Table 2.

## Discussion

In this study, 29.7% adult men had ED. Of those with ED, 12.3 and 9.7% respectively had moderate to severe form. Age and high blood pressure were independent predictors of ED in this setting.

The prevalence of ED in our setting (29.7%) is slightly higher than prevalence observed in Dar es salaam, Tanzania (24%), which also used the international index of erectile function five item version (IIEF-5) scale [12]. The results differ from other-community based study conducted in Southwest Nigeria where prevalence of 48–58.9% was reported by different studies conducted among men aged 30 to 80 years by using international index of erectile function (IIEF) scale [2, 10]. In Egypt, the ED prevalence of 13.4% was obtained using two questions scale on initiation and maintenance of erection for satisfactory sexual intercourse [4]. Differences in age of populations studied, tools in assessing ED and background occurrence of co-morbidities like DM and hypertension may explain the different prevalence observed in SSA studies. However, having one in three men with ED is a serious problem and calls for countries like Tanzania to start investing in men’s sexual and reproductive health in order to achieve the MDG 3 of having healthy lives by 2030.

Age was strongly associated with ED. Our study showed that individuals aged 40–54 and ≥ 55 had higher odds of having ED compared to younger participants aged 18–39 years. Age was also a strong predictor of ED in Ethiopian study where men who aged 40–59 were 6.5 more likely to develop ED and those aged 60 years and above were 7 times more likely to have ED compare to those aged 18–30 years [15]. In spite of comorbidities associated with age, there is decrease in sexual function among men as they age which explains the higher prevalence of ED [16].

The association between smoking and ED had been well established by other authors. The likelihood of having ED was shown even to increase with dose response [17]. Our results are contrary to others as tobacco users had 70% increased chances of having ED compared to non-tobacco users in bivariate but not in multivariate analysis. Researchers have shown that the effect of smoking in erectile dysfunction is through its impact on vascular morphology and physiology and that withdrawal can reverse the damage [18]. We couldn’t find an association of ED and smoking after adjusting for other factors since we had a small proportion of smoker. However, our results are similar to those of Ahmed et al. [19] which showed no association between Tobacco usage and ED after adjusting for confounders [19].

Individuals with diabetes mellitus had higher odds for ED compared to non-diabetic individuals in bivariate but not in multivariate analysis. Results from an Egyptian study done by Zedan et al. [20] reported diabetic individuals had 5 times higher odds of having ED. Also, a study done by Oyelade in Nigeria showed diabetic individuals were 8 times more likely to develop ED [2]. Other studies have shown that diabetic individuals present with ED at a younger age than non-diabetic ones [20]. Diabetes affects vascular system, and this may explain the association with ED. However, lack of association between DM and ED after controlling for confounders in this study may be due to relatively young population (mean age of 39.6 years and very few participants with DM).

In our study participants who were hypertensive had more than 2.6 times higher odds of ED compares to normotensive ones. A study done in west Nigeria also has shown hypertensive individual had a higher prevalence of ED (75%) compared to normal-tensive (56.9%) [11]. Hypertension men have disturbance in vascular endothelia which lead to increase in contraction of vascular smooth muscles [21]. However, some drugs that are used for hypertension management can result into ED [21]. Many men have difficulty in admitting they have ED, and this maybe, worse in African society where masculinity is a norm. As a result, many may stop taking antihypertensive medication, resulting in worse complications like stroke. In many sub Saharan Africa countries including Tanzania, hypertension is becoming a sizeable public health problem [22], hence addressing ED in screening and care services for hypertension is needed to improve adherence to management and overall quality of life.

### Study strengths and limitations

This was a community-based study where participants were selected using probability sampling. The results can be generalized to other men in the municipality. There are limitations also that has to be taken into account when interpreting the results of this study. As a cross-sectional study it is difficult to determine temporal relationship. Men are shy to report problems related to sexual performance, and the tool used to measure ED relied on report from men themselves hence under reporting may have led to underestimation of prevalence of ED. RBG was measured on a single point, and those with impaired RBG advised to go for FBG measurement at nearest facility. This could have underestimate, the results of blood glucose. Use of ward and street leaders in informing the men about the study may have introduced bias. Some men could have thought this is related to government and thus not ready to reveal their experiences related to reproductive health and did not participate. But because total number of men aged 18 and above is not known in selected wards, its, difficult to estimate the direction of bias.

## Conclusion and recommendations

The prevalence of ED is high among men in Moshi municipal as 1 out of 3 men have ED. Age and hypertension were independent predictors of ED. These results point to the need of community awareness and education programs to raise awareness among men about existence of ED problem, its consequence and where they can get advice and care in this setting. Further, health providers taking care of hypertensive and men with DM should be equipped with knowledge and skills on early detection for ED and how to counsel and where to refer patients for help. Given that the problem is substantial, there is a need for future studies (qualitative) to explore understanding and effect of ED on men’s mental health and quality of life.

## Data Availability

The datasets used and analyzed during the current study can be freely available from the corresponding author on reasonable request.

## Abbreviations

BMI: Body Mass Index
BP: Blood pressure
CVD: Cardiovascular Diseases
DM: Diabetes Mellitus
ED: Erectile Dysfunction
HTN: Hypertension
KCMUCo: Kilimanjaro Christian Medical University College
KDC: Kilimanjaro District Council
NCDs: Non-Communicable diseases
RBG: Random Blood Glucose
SDGs: Sustainable Development Goals
SPSS: Statistical Package for Social Sciences
SSA: Sub Saharan Africa
WHO: World Health Organization
